# An economic analysis of a contingency model utilising vaccination for the control of equine influenza in a non-endemic country

**DOI:** 10.1371/journal.pone.0210885

**Published:** 2019-01-24

**Authors:** Sarah M. Rosanowski, Tim E. Carpenter, David Adamson, Chris W. Rogers, Patricia Pearce, Martin Burns, Naomi Cogger

**Affiliations:** 1 Centre for Applied One Health Research and Policy Advice, Jockey Club College of Veterinary Medicine and Life Sciences, City University of Hong Kong, Hong Kong SAR; 2 Epicentre, School of Veterinary Science, Massey University, Palmerston North, New Zealand; 3 The Centre for Global Food and Resources, Faculty of the Professions, The University of Adelaide, Adelaide, South Australia, Australia; 4 Epidemiology and Population Health, The University of Liverpool, Liverpool, United Kingdom; 5 Equine Research Centre, Animal Production and Health Group, School of Veterinary Science, Massey University, Palmerston North, New Zealand; 6 New Zealand Equine Health Association, Wellington, New Zealand; Universita degli Studi di Teramo, ITALY

## Abstract

**Background:**

Equine influenza (EI) is an infectious respiratory disease of horses that has never been reported in New Zealand (NZ). However, the 2007 EI outbreak in Australia, previously EI free, spurred the NZ government and stakeholders into evaluating alternative EI control strategies in order to economically justify any future decision to eradicate or manage EI. To build on the policy debate, this paper presents an epinomic (epidemiologic and economic) modelling approach to evaluate alternative control strategies. An epidemiologic model to determine how alternative EI control strategies influence the distribution of EI. Model results were then input into a cost-benefit analysis framework, to identify the return and feasibility of alternative EI eradication strategies in NZ.

**Methods:**

The article explores nine alternative eradication scenarios and two baseline strategies. The alternative scenarios consisted of three vaccination strategies (suppressive, protective or targeted) starting at three time points to reflect the commercial breeding-cycle. These alternatives were compared to two breeding-cycle adjusted baselines: movement restriction in the breeding season (August to January) or non-breeding season (February to July). The economic loss parameters were incursion response, impact to the commercial racing industry (breeding, sales and racing), horse morbidity and mortality, and compensation to industry participants.

**Results and conclusions:**

Results suggest that the economic viability of the EI eradication programme is dependent on when within the breeding-cycle the EI outbreak occurs. If an outbreak were to occur, the return on each dollar invested for protective or suppressive vaccination strategies would be between NZD$3.67 to NZD$4.89 and between NZD$3.08 to NZD$3.50 in the breeding and non-breeding seasons, respectively. Therefore, protective or suppressive vaccination strategies could be prioritised, regardless of season. As multiple industry stakeholders benefit from these strategies, the study will enable policy development and to better formulate a user-pays eradication programme.

## Introduction

Equine influenza (EI) is a highly contagious respiratory illness affecting all members of the *Equidae* family, including horses, donkeys and mules [1, 2]. While EI is endemic worldwide, New Zealand (NZ), Iceland and Australia are currently EI free [3]. However, in August 2007, an outbreak of EI occurred in the naïve equine population in Australia [4, 5]. During that outbreak, EI spread rapidly between horses and horse properties, resulting with the Australian Government spending AUD$100 million to eradicate EI and allocating an additional AUD$260 million to support the industry [6]. The outbreak was eradicated through the implementation of movement restrictions, the cancellation of equine events such as race meetings and sport horse competitions, and by a pro-active campaign of vaccinating at-risk horses [4]. However, the true cost of the Australian EI outbreak remains unknown as the disruption to the normal equine activities has not been fully quantified [7].

The Australian EI outbreak provided a stimulus for NZ decision makers to consider the possible implications for the NZ equine industry. Horse owners, industry and the government were concerned with the potential economic and social implications EI could have in NZ. To justify and prioritise strategies for EI control, an epidemiologic model was developed to provide an EI simulation platform to examine how EI may spread through NZ [8]. In order to examine the rationale for a national eradication campaign for EI in NZ, epidemiologic modelling of control strategies has been extended by adapting findings into an economic framework. This study extends the epidemiologic modelling by [8] conducting a cost-benefit analysis (CBA) to determine if a national eradication campaign in NZ could be justified economically. Additionally, whilst annual seasonality was not included in the original epidemiologic model, here it was hypothesized that EI would be most economically disruptive during the commercial breeding season (August to January). This hypothesis was based on the commercial incentive to produce horses born as close to the official Southern Hemisphere birthdate of the 1^st^ of August, to produce a physically mature product for yearling sales the following year [9–13]. Consequently, the economic model allows for the impact of the seasonally adjusted baseline control strategies to be compared to alternative control strategies, including vaccination, based on the non-seasonally adjusted outputs of previously published epidemiologic model scenarios [8].

## Materials and methods

Evidenced based policy is generated by merging scientific findings into economic platforms to help illustrate the consequences of real trade-offs, in order to inform social debate. This paper combines an epidemiologic and economic model in an epinomic framework to rationalise outcomes from alternative policy choices. Findings from the updated epidemiologic model are used to shape the EI scenarios and provide data to justify the assumptions used in the economic platform. This two stage modelling process then helps provide the necessary scientific and economic rigour required to evaluate changes in economic welfare from evidenced based policy [14].

### Study population

Horses in NZ fall within two major categories, those in the racing industries and non-racing horses. The racehorses are registered purebred animals and include those used for racing and breeding. Due to international requirements (Stud book and racing regulation) and domestic regulations focusing on integrity of racing and gambling, there are robust data on the size and scope of the Thoroughbred (horses racing under saddle on the flat or over jumps) and Standardbred (horses, often gaited, that race ‘in harness’) racing and breeding industries. In 2010 there was a total of 25,623 animals listed in the respective studbooks, including horses in active race training (racehorses) and breeding animals; mares, stallions or youngstock [15–17].

In contrast, non-racehorses maybe be purebred or cross-bred and used for a range of activities including sports, recreation, as companion animals, or for work. Quantifying the size of the non-racing population is problematical, as the NZ government does not require the registration of all non-racing horses. However, an estimation of the number of non-race horses was obtained by combining two data sources and then subtracting the total number of racing horses. The first data source was a census of agricultural businesses [18] and the second a rural database, AgriBase [19]. A census of agricultural businesses was conducted in 2012, which identified 56,878 horses kept on registered agricultural business properties [18]. In addition, many properties keeping non-race horses are listed in AgriBase [19] and in 2010 there were 18,329 properties listed in AgriBase as keeping horses. While AgriBase also contains information about the number of horses on a property, previous studies have identified inaccuracies in these data [20]. As such, the number of non-racing horses was estimated at 31,255. Based on the total number of racing and non-racing horses and the number of properties, the mean number of horses was estimated at 3.1 per property.

### Epidemiologic framework

The InterSpread Plus model, described previously by Stevenson, Sanson [21], was used to simulate EI control strategies in NZ [8]. InterSpread Plus is a stochastic, spatio-temporal, state-transition model, with susceptible, latent, infected, and recovered (SLIR) states. The unit of interest is the property-level, with time steps for each day of the outbreak, and property location based on coordinate data included in the model.

For the simulated outbreak, the level of pre-detection surveillance was set so that EI was detected within a similar timeframe to the Australian outbreak in 2007 [4, 22]. Using the AgriBase database, five types of horse property (general horse, Thoroughbred training, Thoroughbred breeding, Standardbred training, and Standardbred breeding) plus Thoroughbred and Standardbred racecourses were defined, with location noted, based on coordinate data. The InterSpread Plus model was parameterised with horse movement data, depending on property type [9, 23, 24]. Additional parameters, including infectivity of EI, between property spread of EI (including local spread), contact tracing, surveillance and veterinary resources for vaccination, were simulated. Based on previous studies [25, 26], a total of 90 iterations of each scenario were run and the iteration was run until there were no new cases of EI or for a maximum of 180 days. All scenarios were seeded in a Thoroughbred stud farm in the Waikato, based on the destination of horses imported into NZ [27] and the density of commercial and non-commercial horse properties in that area [9, 20, 28].

The baseline control strategy included movement restriction beginning on the first day of official EI detection and continued for the remainder of the simulation. Three alternative control strategies of emergency vaccination were investigated: suppressive, protective and targeted. These strategies were implemented over and above movement restriction and started 14 days after the official detection of EI in the population. The suppressive strategy involved vaccinating all susceptible animals on properties within a 3 kilometre radius around an infected property. The protective strategy involved ring vaccination. The inner ring of vaccination started 7 kilometres from the infected property and included all horse properties up to the outer ring, 10 kilometres from the infected property (i.e. a 7 to 10 kilometre ring). Targeted vaccination involved the vaccination of all breeding and racing properties within 20 kilometres of an infected property. Due to the location of the veterinary personnel and the density of horse properties, personnel resources for vaccination were prioritised. This prioritisation of resources was modelled with the vaccination of five properties in the Auckland and Waikato region to one property vaccinated in the rest of NZ.

The output of the model included the median and interquartile range of epidemic duration in days, number of infected properties, and for the alternative strategies, number of vaccinated properties [8]. Median duration was 88, 92, 136 and 178 days for suppressive, protective, targeted and baseline strategies, respectively. Median number of infected properties ranged from 793 for the suppressive strategy, to 3,136 for the baseline strategy. Median number of vaccinated properties was 1,653, 2,726 and 4,502 for targeted, suppressive and protective strategies, respectively.

### Economic framework

Cost-benefit analysis (CBA) provides a platform to evaluate the costs and benefits from alternative choices. In this case the CBA is used to evaluate the rationality of action when dealing with an exotic disease within a naïve population. Consequently, all scenarios assume that individuals will comply with movement restriction and quarantine instruction. Therefore, the take no vaccination action (‘without’) provides the scenario where decision makers take no vaccination action but implement the minimum control strategy: movement restriction, contact tracing and active surveillance against EI. It is against this benchmark that all other action (‘with’), is compared. Therefore, the benefits from intervention are derived from its actions against EI, including eradiation, management, vaccination etc. Unlike a traditional CBA, this analysis has only considered all cost and benefits to accrue for the duration of the outbreak, consequently a discount rate is not used to evaluate multiple year costs and benefits. All data have been placed into 2017 monetary values.

Within the framework, cost parameters include the cost of an incursion management response, including vaccination (labour costs and material costs). While benefits are derived from any reduction in the number of animals lost or infected (i.e. minimising the impact on industry) and the reduction in costs associated with shortening the outbreak duration, including incursion response other than vaccination, morbidity and mortality, compensation to trainers and breeding, sales and racing income. Non-racing horses were considered in the incursion response, and morbidity and mortality sections of the economic framework, but were not considered with regards to compensation to trainers, breeding, sales and racing (competitive) income as these activities were considered to be non-commercial in the non-racing industry.

Key to the costs of an EI outbreak is understanding the relationship between disease prevalence and the commercial breeding season, henceforth called the breeding cycle, and commences on the 1^st^ of August. Using the control strategies generated through epidemiologic modelling, economic scenarios were generated to adjust for the breeding-cycle. If an outbreak were to occur between February and July (non-breeding season), little disruption to the breeding-cycle was assumed, due to lack of horse movement to and from stud farms and sales activities [9]. Given this, two ‘without’ baseline scenarios were simulated: movement restriction on the first day of official detection for i) an outbreak beginning at the start of the breeding season in August (Baseline 1) and ii) an outbreak beginning outside of the breeding season (February, Baseline 2), due to the median duration of an outbreak under a movement restriction strategy (178 days, inter quartile range (IQR) 150 to 180 days) [8]. During the breeding season (August to January), one-third of the breeding activity was generated by stud farms between August and October and two-thirds between November and January [9]. Consequently, for each of the three alternative control strategies identified in epidemiologic modelling, scenarios were simulated, adjusted by breeding-cycle for outbreaks beginning at the start of the breeding season (August to October), mid-breeding season (November to January) and in the non-breeding season (February to July). Scenarios were weighted to address the differences in activity levels. A breeding-cycle weighting of 33% or 67% was applied to the income generated through mares served (stud fees) and horse sales for August to October and November to January, respectively, with no impact in the non-breeding period (0% change). The impact of season on scenarios <90 days duration were calculated based on the percentage decrease in sales or breeding, multiplied by the day adjusted breeding cycle weighting (33% or 67%). The impact of an outbreak when scenarios were ≥90 days duration were calculated based on the breeding cycle weighting at the start of the breeding season (33% or 67%) and then the day adjusted breeding cycle weighting of 67% or 33% for outbreaks at the start of the breeding season and mid-season, respectively. By adjusting each alternative strategy by breeding-cycle, a total of nine strategies were created; six to compare with Baseline 1 and three to compare with Baseline 2 (Table 1). Due to the similarities between the Australian and NZ industries (and the nature of the formerly naïve population), Australian data for alternative management options were used for the simulations reported here. All Australian loss data in dollars have been adjusted for inflation [29] and converted to NZ dollars ($AUD1 to NZD$1.06, July 2017). Consequently, all dollar values reported within the rest of the article are in 2017 NZ dollars.

**Table 1:** Description of the breeding season adjusted baseline and alternative control strategies for an equine influenza outbreak in New Zealand.

Scenario ^a^	Breeding cycle	Vaccination	Impact on income generated through breeding (range)
Breeding season			
Baseline 1	August to January	None	100%
Suppressive	August to October	3km radius^b^	32% (29%-46%)
Protective	August to October	7 to 10 km ring^c^	34% (29%-43%)
Targeted	August to October	20 km radius^d^	67% (51%-96%)
Suppressive	November to January	3km radius^b^	66% (58%-73%)
Protective	November to January	7 to 10 km ring^c^	68% (59%-72%)
Targeted	November to January	20 km radius^d^	84% (76%-98%)
Non-breeding season			
Baseline 2	February to July	None	0%
Suppressive	February to July	3km radius^b^	0%
Protective	February to July	7 to 10 km ring^c^	0%
Targeted	February to July	20 km radius^d^	0%

^a^ For all scenarios, movement restriction was applied from the first day of EI detection.

^b^ Vaccination in a 3 km radius of an infected property starting 14 days after official detection.

^c^ Vaccination in a 7 to 10 km ring around an infected property starting 14 days after official detection.

^d^ Vaccination of all properties involved in the racing industry (training and breeding) in a 20 km radius of an infected property starting 14 days after official detection.

#### Industry income

Critical to the development of an economic analysis is the estimation of the income generated by the equine industry. Income generated by different sectors within the equine, specifically racing, industry was estimated, and income can be derived from three sources breeding, sales, and racing activity (Table 2).

**Table 2:** Sources of data used for the economic analysis to determine the impact of an equine influenza outbreak on the New Zealand equine industry. Values in NZD unless specified and unadjusted for inflation.

Type	Parameter	Source	Value
**Income generation**	Stakes for Thoroughbred races	New Zealand Thoroughbred Racing Fact book 2010 [28]	$49.6 million
	Stakes for Standardbred races	Harness Racing New Zealand (2012/13 season) (www.hrnz.co.nz)	$23.5 million
	Number of Thoroughbred broodmares, foals, stallions, stud fee per stallion, % live foals	Description of the Thoroughbred breeding industry (2008–2012) [16], [15]	$106.6 million*
	Number of Standardbred broodmares, foals, stallions, % live foals	Standardbred Breeders Association stud book– 2013 (www.harnessracing.co.nz)	$18.6 million*
	Sales data		
	Thoroughbreds—yearlings, weanlings, mixed, ready to race	New Zealand Bloodstock website (2012/2013 data) (www.nzb.co.nz/sales)	$95.3 million
	Standardbreds—yearlings	PPG Wrightsons website (2013 data) (www.standardbred.co.nz/Sales/Results)	$9.6 million
**Disease parameters**	Duration of the outbreak, number of infected properties, number of vaccinated properties	Epidemiologic modelling [8]	
**Industry impact**	Thoroughbred breeding	Australian Racing Fact Book 2007/08 [37]	25%
	Thoroughbred sales	Australian Racing Fact Book 2007/08 [37]	14%
	Standardbred breeding	New Zealand Standardbred Breeders Association Studbook, 2013 (www.harnessracing.co.nz) Harness Racing Australia On-line Annual Statistics (http://www.harness.org.au/hra/annual/public/stats.htm)	39%
	Standardbred sales	Australian Harness Racing Council and Harness Racing Australia Sales data, 2007, 2008, 2009, 2010, 2011 (http://www.harness.org.au/)	14%
	Compensation	Commercial Horse Assistance Programme [32]	AUD$60 Thoroughbred AUD$20 Standardbred

* Values from Bolwell, Rogers [15]

Total income generated from the breeding component was calculated by multiplying the stud fee for each standing stallion and by the number of mares served by that stallion, with the total summed to include all stallions. According to Bolwell, Rogers (15), during the 2012/2013 season, there were 94 Thoroughbred stallions and 90 Standardbred stallions standing, and 5,202 Thoroughbred and 3,062 Standardbred mares were served. The mean service fee of the Thoroughbred industry and the Standardbred industry was $21,374 and $6,333, respectively. Total breeding income (ie = stud fees) was $130.6 million, of which $11.2 million was derived from Thoroughbred breeding and $19.4 million from Standardbred breeding.

Sales income was obtained from yearling, weanling and mixed aged sales, plus ready-to-run sales for Thoroughbreds (Standardbreds do not hold ready-to-run sales). Number of lots sold at sales, and the aggregate sales price of each sale were collated from industry websites for 2012 and 2013 (www.nzb.co.nz/sales; www.standardbred.co.nz/Sales/Results). From this approach, it is estimated that total sales income of Standardbred sales was $10.0 million, and $99.4 million was generated from Thoroughbred sales.

For Thoroughbred and Standardbred racing, income was determined based on the stakes earnings for the year for each racing code [16], with stakes of $49.6 million and $23.5 million for Thoroughbred and Standardbred racing, respectively. Basic earnings by jockeys for riding a horse in a race were estimated at $133 per start [16]. Income for a Standardbred (i.e. harness racing) driver per starter at a race meeting was estimated as half the jockey fee ($66). Only one-third of starters in harness races were considered as fee earning drives, as in the remaining starters the driver was the trainer of the horse and would not have been paid for this service [17]. Between 2005 and 2013, the median number of Thoroughbred and Standardbred horses actively participating in racing per annum was 5,853 and 3,628, respectively. During that time, Thoroughbred racehorses engaged in 32,586 race starts per year and Standardbred racehorses engaged in 31,135 race starts per year [15, 17].

#### Income generated by breeding, sales and racing

In the absence of an EI outbreak, it was estimated a total of $324.33 million was generated through racing industry related activity in 2017 season (Table 3). Thoroughbred sales generated $99.4 million and Standardbred sales $10.0 million. Stud fees generated $111.2 and $19.4 million for Thoroughbred and Standardbred breeders, respectively. A total of $54.9 million and $24.5 million in stakes were generated by Thoroughbred and Standardbred racing, respectively. Based on the number of racing starts, the total income to jockeys was $4.3 million and $0.6 million to drivers. Racing income did not include additional income generated by trainers, jockeys or drivers through stakes winnings. Income generated by the non-racing (competition and pleasure) sectors was not considered due to the difficulty quantifying the economic contribution of horses kept for non-racing purposes. Table 3 summarises the sources of income-generation data included in the study.

**Table 3:** An example of the input values and losses associated with the eradication of equine influenza in New Zealand under the suppressive vaccination strategy in the breeding season (August to October). The duration of the outbreak, number of infected and vaccinated properties determined through simulation modelling.

Variable	Input	Total loss (millions)	Loss parameter
**(1) Incursion Response**		$44.5	
	Per day response costs		$250,000
	# days active management		70
	Per day response costs		$125,000
	# days of active management		18
**CV—Additional cost of alternative strategies (Vaccination Cost)**		$24.8	
	Per horse vaccination costs		$210
	# of properties vaccinated		2,726
	# horses vaccinated		8,451
	Labour cost per person/day		$1,280
	# of groups		68
	# of people per group		3
	Other costs (/person/day)		$150
**(2) Morbidity and Mortality**		$2.9	
	Cost per property for veterinary treatment		$1,500
	# of infected properties		793
	Cost per horse for mortality		$14,200
	# of infected horses		2,458
	Mortality (5% of all horses infected)		122.9
**(3) Compensation**		$45.2	
	Per day payment per Thoroughbred horses in work		$71
	# Thoroughbred horses in work		5,853
	Per day payment per Harness horses in work		$26
	# Harness horses in work		3,628
	# of days of compensation		88
**(4) Industry impact**		$36.6	
**Breeding and sales**		$16.3	
	Importance of season (%)		32
**Thoroughbred**	Sales loss	$4.5	
	Percentage change in sales		14
	Percentage of season affected (%)		4.5
	Stud fees loss	$9.0	
	Percentage of mares not served due to outbreak		25
	Percentage of season affected		8.1
**Standardbred**	Sales loss	$0.4	
	Percentage change in sales		13.5
	Percentage of season affected		4.4
	Stud fees loss	$2.4	
	Percentage of mares not served due to outbreak		39
	Percentage of season affected		12.6
**Racing and training**		$20.3	
**Thoroughbred**	Races cancelled	$13.3	
	# per day		0.9
	Stakes per race meeting		$167,406
**Standardbred**	Races cancelled	$5.9	
	# per day		0.73
	Stakes per race meeting		$92,493
**Training losses**		$1.0	
	Riding fee for jockeys		$133
	# of starts per day		89
	Fee for drivers		$66
	% of starts where driver also the trainer		0.7
	# of starts per day		26
**Total losses associated with an EI outbreak**		**$129.2**	

#### Income loss

Within the CBA, estimations of the income loss parameters in the event of an EI outbreak were required. Four areas where there would be a loss due to an EI outbreak included incursion response, horse morbidity and mortality, compensation paid to racehorse trainers and the impact on the racing and breeding industries. Income information is presented in Table 2.

#### 1) Incursion response

Incursion response was calculated as the cost of movement restriction, surveillance, contact tracing, and organisation and administration. An approximate spend on control of $500,000 per day during the EI outbreak in Australia in 2007 [4], which was all-inclusive (movement restriction, surveillance, contact tracing and vaccination) and included two states with a landmass of 280,000 km^2^, 10,000 properties, and 140,000 horses were vaccinated as part of the incursion response [B. Cowled, cited in 6]. While the landmass in NZ is comparable to New South Wales (NSW) and Queensland, the population of horses is smaller, and consequently any predicted outbreaks are also smaller than what occurred in 2007 and the predicted number of infected properties and vaccinated horses is lower than what occurred in Australia [8]. Therefore, the cost of an incursion response excluding vaccination was estimated at $250,000 per day for the first 70 days of the outbreak, based on the peak of the epidemic curve of 55 days under the baseline epidemiologic model [8], plus two further weeks of heightened response. Subsequent to this, the cost of implementing an incursion response was estimated at $125,000 per day until the end of the outbreak.

Separately, vaccination cost (CV) was calculated at $210 per horse for microchipping and two vaccinations, labour costs at $160 per hour, for teams of three people (a veterinarian, a recorder and a horse handler) and a sundry cost per person of $150 per day for accommodation, travel and food. The number of teams available for vaccination was determined based on the number of veterinarians registered with the New Zealand Veterinary Association as Equine Practitioners (n = 270) (http://nzeva.nzva.org.nz/home).

#### 2) Morbidity and mortality

Case specific mortality associated with EI was determined to be 5%, which was estimated based on a survey of horse property owners in one region of NSW [30]. The morbidity associated with equine influenza was estimated based on proportions of seropositive horses on infected properties after the Australian outbreak, with the proportion of seropositive horses on the property ranging between 9% and 100% (median 100%; mean 96.8%) [31]. The proportion of horses that were seropositive varied by the number of horses on the farm, with larger farms (>20 horses) having a lower risk of all horses being seropositive. As such, it was estimated that on average each property owner spent $1,500 (AU$1,414) on veterinary costs for all horses on their property [30]. Of the 5% of horses that died, the mean price (value) per horse was determined to be $14,200 (AU$13,515) [30]. As NZ and Australia have similar equine populations, in terms of previous exposure to EI and vaccination status, it was assumed that the mortality and morbidity rates would be the same between the two populations.

#### 1) Compensation

In Australia, the Commercial Horse Assistance Programme (CHAP) was set up to provide payments to owners and trainers of horses that were used for commercial or income earning purposes [32]. Assistance per day per horse was AU$68 per Thoroughbred racehorse and AU$25 for a Standardbred racehorse. In the model, compensation was modelled at $71 and $26 per day for Thoroughbreds and Standardbreds, respectively.

#### 1) Industry impact

For each sector of the equine industry, the impact on generated income due to an EI outbreak was determined. The impact on breeding and sales was extrapolated from Australian data, with the impact on the breeding sector including the number of mare services and the live foal reports from the 2007/2008 breeding season. Sources of data for this can be found in Table 3. Based on those data, it was estimated that the NSW and Queensland (QLD) Thoroughbred and Standardbred breeding sectors were affected by a drop in mare services of 8% (all Australia data) and 29%, respectively. Subsequently, the number of foals born dropped by 17% and 10%, in the Thoroughbred and Standardbred sectors, respectively. Based on this, the total impact on breeding was 25% and 39% for Thoroughbred and Standardbred breeding income, respectively. In total, 14.0% fewer Thoroughbred and 13.5% fewer Standardbred horses were sold in the post EI epidemic year (2008) compared to the previous year. Standardbred sales data were from NSW and QLD only. The model did not account for the complete loss of sales revenue due to the cancellation of sales.

The impact of EI on racing income was modelled on a daily basis by calculating the income from stakes per race meeting multiplied by the number of race meetings for the duration of the outbreak. The median number of race meetings per year held for Thoroughbreds was 328 and 265 for Standardbreds over the last 7 years [16]. The loss of income for jockeys and drivers through the cancellation of races was calculated. The loss of income to jockeys was calculated by the mean number of starters per day (total number of starters/365 days) multiplied by the ride fee multiplied by the duration of the outbreak. The income for a driver per starter at a race meeting was estimated as half the jockey fee ($66). In total, 70% of the drivers in harness races were not fee earning, because they both trained and drove the horse in the race [17].

### Income loss, benefit, benefit-cost ratio (B/C ratio) and stakeholder benefit

Income loss, defined as total income lost due to an outbreak of EI, was described for each scenario. The benefit (B), total cost of the baseline strategy minus losses and costs that were avoided (not realized) due to the alternative strategy and CV in that scenario were calculated for the nine alternative scenarios. Stakeholder benefit for each element of the control strategy: incursion response (minus the cost (CV)), morbidity and mortality, compensation and industry impact, with industry impact stratified by breeding and racing, was described. An example of the inputs and results of the epinomic model is provided in Table 3. For this analysis, it has been assumed that all costs and all benefits occur in a single year, therefore the values are not discounted. This is a simplification of future benefits, but this assumption was used to help illustrate to the public the benefits from eradicating EI as quickly as possible.

Net benefit (NB) was calculated by comparing the total income loss of the baseline strategy in either the breeding or non-breeding season, to each of the alternative strategies. Net benefit was used to calculate the feasibility of the alternative scenarios. Feasibility was considered to be achieved if the NB was greater than $0. The B/C ratio was used to determine if a control strategy was able to provide a return on the investment, with ratios greater than 1 being above the breakeven point. The B/C ratio was calculated as the benefits (B) of the alternative control strategy divided by the additional costs of vaccination (CV).

For each scenario, the range for each benefit (B), vaccination cost (CV), net benefit (NB) and B/C ratios were calculated based on the IQR of epidemiologic model values: epidemic duration, number of infected properties, and number of vaccinated properties.

### Sensitivity analysis

A sensitivity analysis was performed to evaluate responsiveness of the results to changes in the B/C ratio and NB. Four different scenarios were examined. The first alternative scenario was that the total horse population was increased to a maximum estimate of 120,000 horses [33]. This population estimation was based on the number of horses listed in the AgriBase database. This estimation was considered to be the upper limit of the size of the NZ horse population; however, inaccuracies in the information recorded in the AgriBase database have been identified previously [20]. The second alternative scenario was that the percentage mortality was increased to 10% compared to 5% [30]. This assumption was based the potential for variation in virulence between EI strains. A further decrease in breeding and sales income by 20%, to reflect differences in the Australian and NZ commercial breeding industries.

Two sensitivity analyses focused specifically on the breeding industry. Firstly, a further 20% decrease in breeding income, to 45% and 59% in Thoroughbreds and Standardbreds, respectively. Secondly, a further 20% decrease on sales income, to 34% and 33.5% for Thoroughbreds and Standardbreds, respectively. Both analyses were applied to the baseline and three vaccination strategies, with outbreaks occurring in the breeding season only. In Australia, sales of horses reduced by 14.0% for Thoroughbreds and 13.5% for Standardbreds, and breeding by 8% for Thoroughbreds and 29% for Standardbreds. Most likely, this is an underestimate of the effect of an EI outbreak in NZ. In Australia, control measures were only applied to the affected states, NSW and Queensland and unaffected states could continue business activities almost as normal. The percentage change in the NB and the B/C ratio under each scenario were calculated.

## Results

The overall loss was highest under the baseline strategy for an outbreak in the breeding season ($225.5 million) and in the non-breeding season ($174.9 million) (Table 4). The additional cost of vaccination (CV) was $24.8 million, $27.0 million and $36.6 million for suppressive, protective and targeted vaccination strategies, respectively. The NB of the suppressive strategy was $96.3 million if it were implemented between August and October, and $62.1 million if it were implemented in the non-breeding season. The net benefit of the protective strategy was $89.4 million if it were implemented between August and October, and $58.4 million if it were implemented in the non-breeding season. The net benefit of the targeted strategy was $20.1 million if it were implemented between August and October, and $3.5 million if it were implemented in the non-breeding season.

**Table 4:** The estimated cost of vaccination, and losses prevented (benefits) for the nine alternative control scenarios, compared to baseline strategies applied in either the breeding season (August to January) or non-breeding season (February to July). The duration of the outbreak, number of infected and vaccinated properties determined through simulation modelling.

Breeding-cycle	Strategy	Income loss (range)	Benefit (range)	Net Benefit (range)	Cost of vaccination (range)	B/C (range)^d^
**Breeding season**	Baseline	$225.5 ($200.2, $228.1)				
**August to October**	Suppressive^a^	$129.2 ($114.4, $159.4)	$121.1 ($62.5, $143.9)	$96.3 ($40.7, $113.7)	$24.8 ($21.7, $30.2)	4.89 (2.87–4.77)
	Protective^b^	$136.1 ($116.3, $157.2)	$116.4 ($65.5, $142.7)	$89.4 ($42.9, $111.9)	$27.0 ($22.6, $30.8)	4.31 (2.9–4.63)
	Targeted^c^	$205.4 ($171.6, $264.9)	$56.7 ($-34.0, $103.4)	$20.1 ($-64.8, $56.6)	$36.6 ($30.8, $46.8)	1.55 (-1.11–2.21)
**November to January**	Suppressive^a^	$146 ($129.3, $173.4)	$104.3 ($48.5, $129)	$79.5 ($26.8, $98.8)	$24.8 ($21.7, $30.2)	4.21 (2.23–4.27)
	Protective^b^	$153.4 ($131.4, $171.8)	$99.1 ($51.0, $127.6)	$72.1 ($28.4, $96.8)	$27 ($22.6, $30.8)	3.67 (2.26–4.14)
	Targeted^c^	$213.9 ($184.2, $265.9)	$48.3 ($-35.0, $90.8)	$11.7 ($-65.7, $43.9)	$36.6 ($30.8, $46.8)	1.32 (-1.14–1.94)
**Non-breeding season**	Baseline	$174.9 ($149.5, $177.4)				
**February to July**	Suppressive^a^	$112.8 ($99.9, $136.3)	$86.9 ($34.9, $107.7)	$62.1 ($13.2, $77.5)	$24.8 ($21.7, $30.2)	3.50 (1.61–3.57)
	Protective^b^	$118.7 ($101.6, $135.2)	$83.2 ($36.9, $106.7)	$56.2 ($14.3, $75.8)	$27 ($22.6, $30.8)	3.08 (1.63–3.46)
	Targeted^c^	$171.4 ($145.8, $216.2)	$40.1 ($-36.0, $78.4)	$3.5 ($-66.7, $31.6)	$36.6 ($30.8, $46.8)	1.10 (-1.17–1.67)

All values in the table per million New Zealand dollars

^a^ Vaccination in a 3 km radius of an infected property starting 14 days after official detection, strategy included movement restriction starting on the first day of EI detection.

^b^ Vaccination in a 7 to 10 km ring around an infected property starting 14 days after official detection, strategy included movement restriction starting on the first day of EI detection.

^c^ Vaccination of all properties involved in the racing industry (training and breeding) in a 20 km radius of an infected property starting 14 days after official detection, strategy included movement restriction starting on the first day of EI detection.

^d^ B/C is the benefit cost ratio

### Cost-benefit analysis

For suppressive and protective scenarios applied in the breeding season, the B/C ratio ranged from 3.67 to 4.89 (Table 4). In an early breeding season outbreak, a suppressive vaccination strategy would return $4.89 for every dollar invested in control, compared to $4.31 for a protective strategy. In a late breeding season outbreak, a suppressive strategy would return $4.21 and a protective strategy $3.67 per dollar invested in control. The targeted vaccination strategy had the lowest B/C ratio at between $1.10 in the non-breeding season and $1.55 early in the breeding season.

### Stakeholder benefit

The reduced loss under each of the alternative scenarios is presented in Table 5. The loss of an incursion response during an EI outbreak under the baseline strategy was $31.0 million. The reduced loss, through vaccination, was $11.3 million, $10.8 million and $5.3 million, for suppressive, protective and targeted strategies, respectively. In the baseline strategy, the loss generated through providing compensation to racehorse trainers was the highest ($91.3 million, regardless of season). For all scenarios, compensation provided the greatest reduction in losses compared to other industry areas at $45.2 million, $47.2 million and $69.8 million for suppressive, protective and targeted strategies, respectively. Regardless of season, mortality losses were $6.9 million, $1.7 million, $1.9 million, and $4.7 million for baseline, suppressive, protective and targeted strategies, respectively. Under a suppressive strategy, the loss associated with the breeding and sales of horses was reduced by $33.2 million and $16.3 million for outbreaks occurring early and mid-breeding season, respectively, compared to the baseline strategy.

**Table 5:** The estimated losses and reduced losses to parameters within the economic model; incursion response, horse mortality and morbidity, breeding income, racing income and compensation. The duration of the outbreak, number of infected and vaccinated properties determined through simulation modelling.

Breeding cycle	Scenario	Incursion response		Mortality and morbidity		Breeding		Racing		Compensation	
		Losses (cost of vaccination)	Reduced cost	Losses (cost of mortality)	Reduced cost	Losses (cost of stud fees)	Reduced cost	Losses (Cost of lost race stakes)	Reduced losses	Losses	Reduced losses
Breeding season	Baseline^a^	31.0		11.6 (6.9)		50.6 (33.9)		41.0 (35.8)		91.3	
August to October	Suppressive^b^	44.5 (24.8)	11.3	2.9 (1.7)	8.7	16.3 (11.4)	34.3	20.2 (17.7)	20.7	45.2	46.2
	Protective^c^	47.2 (27.0)	10.8	3.1 (1.9)	8.5	17.4 (12.1)	33.2	21.2 (18.5)	19.8	47.2	44.1
	Targeted^d^	62.4 (36.6)	5.3	7.9 (4.7)	3.7	34.0 (23.8)	16.6	31.3 (27.3)	9.7	69.8	21.6
November to January	Suppressive	44.5 (24.8)	11.3	2.9 (1.7)	8.7	33.2 (23.1)	17.5	20.2 (17.7)	20.7	45.2	46.2
	Protective	47.2 (27.0)	10.8	3.1 (1.9)	8.5	34.7 (24.2)	16.0	21.2 (18.5)	19.8	47.2	44.1
	Targeted	62.4 (36.3)	5.3	7.9 (4.7)	3.7	42.5 (29.7)	8.2	31.3 (27.3)	9.7	69.8	21.6
Non-breeding season	Baseline	31.0		11.6 (6.9)		0.0 (0.0)		40.5 (35.8)		91.3	
February to July	Suppressive	44.5 (24.8)	11.3	2.9 (1.7)	8.7	0.0 (0.0)	0.0	20.2 (17.7)	20.7	45.2	46.2
	Protective	47.2 (27.0)	10.8	3.1 (1.9)	8.5	0.0 (0.0)	0.0	21.2 (18.5)	19.8	47.2	44.1
	Targeted	62.4 (36.6)	5.3	7.9 (4.7)	3.7	0.0 (0.0)	0.0	31.3 (27.3)	9.7	69.8	21.6

All values presented per million New Zealand dollars

^a^ Movement restriction starting on the first day of EI detection.

^b^Vaccination in a 3 km radius of an infected property starting 14 days after official detection, strategy included movement restriction starting on the first day of EI detection.

^c^ Vaccination in a 7 to 10 km ring around an infected property starting 14 days after official detection, strategy included movement restriction starting on the first day of EI detection.

^d^ Vaccination of all properties involved in the racing industry (training and breeding) in a 20 km radius of an infected property starting 14 days after official detection, strategy included movement restriction starting on the first day of EI detection.

### Sensitivity analysis

Results of the sensitivity analysis are summarised in Table 6. A further 20% decrease in breeding income had the greatest impact on the overall losses in the baseline, suppressive and protective scenarios starting in August. For the baseline strategy, a further decrease in breeding income created the greatest change in overall loss at 10.4% ($26.1 million), increasing the overall loss to $251.6 million. For the suppressive and protective strategies, the overall losses rose by 6.1% ($8.4 million) and 5.9% ($8.5 million), respectively. Increasing the size of the equine population to 120,000 had the greatest change on the overall losses for the targeted strategy, with an increase of 3.0% ($6.4 million).

**Table 6:** The overall loss, benefit cost ratios (B/C) and percentage change compared to the original economic models for four sensitivity models; Increasing the horse population size, increasing mortality, decreasing sales income and decreasing breeding income for scenarios. Sensitivity analysis was conducted only for the first three months of the breeding season (August to October). The duration of the outbreak, number of infected and vaccinated properties determined through simulation modelling.

Control Strategy	Maximum population size (120,000 horses)		Increased mortality (10%)		Further 20% decrease in sales income		Further 20% decrease in breeding income	
	Change in loss (% change)	B/C ratio (% change)	Change in loss (% change)	B/C ratio (% change)	Change in loss (% change)	B/C ratio (% change)	Change in loss (% change)	B/C ratio (% change)
**Baseline**^a^	7.6 (3.2)		6.9 (3.0)		26.1 (10.4)		7.1 (3.1)	
**Suppressive**^b^	3.9 (2.9)	4.74 (-3.2)	1.7 (1.3)	5.09 (3.9)	8.4 (6.1)	5.60 (12.7)	7.1 (5.2)	4.89 (0)
**Protective**^c^	4.9 (3.5)	4.21 (-2.4)	1.5 (1.1)	5.30 (18.7)	8.5 (5.9)	4.50 (4.2)	7.1 (4.9)	3.94 (-9.4)
**Targeted**^d^	6.4 (3.0)	1.73 (10.4)	-4.1 (-2.0)	2.66 (41.7)	4.2 (2.0)	0.64 (-142.2)	2.1 (1.0)	1.51 (-2.6)

All percentage change values are compared to the original strategy

^a^ Movement restriction starting on the first day of EI detection.

^b^ Vaccination in a 3 km radius of an infected property starting 14 days after official detection, strategy included movement restriction starting on the first day of EI detection.

^c^ Vaccination in a 7 to 10 km ring around an infected property starting 14 days after official detection, strategy included movement restriction starting on the first day of EI detection.

^d^ Vaccination of all properties involved in the racing industry (training and breeding) in a 20 km radius of an infected property starting 14 days after official detection, strategy included movement restriction starting on the first day of EI detection.

A further 10% increase in mortality had the least impact on the overall losses in the baseline, suppressive and protective scenarios starting in August. A further increase in mortality increased the overall loss by 3.0% ($6.9 million), 1.3% ($1.7 million), and 1.1% ($1.5 million) for the baseline, suppressive and protective strategies, respectively. Increasing the case specific mortality rate reduced the overall loss for the targeted strategy by $4.1 million (2.0%).

The greatest change in the B/C ratio for the suppressive strategy was with a further 20% decrease in breeding income with a 12.7% increase to $5.60 per $1.00 spent on control, while an increase in the population size would reduce the B/C by 3.2% to $4.74. The greatest change in the B/C ratio for the protective strategy was a 10% increase in mortality, with an 18.7% increase to $5.30 per $1.00 spent on control, while decreasing the sales income would reduce the B/C ratio by 9.4% to $3.94.

The B/C ratio was most sensitive to any change examined under the targeted control strategy, with changes of 142.2%, 41.7%, 10.4% and 2.6% for the decreasing in breeding, increased mortality, increased population size, and decrease sales analyses, respectively. For the decrease in breeding income analysis, the B/C ratio was $0.64 per $1.00 spent on control, the only strategy that did not provide a favourable return on every dollar invested.

## Discussion

This study attempts to quantify the economic impact of an EI outbreak on the NZ equine industry and provide a rationale for vaccination in the face of an outbreak. The CBA conducted here, supports the implementation of suppressive or protective vaccination, particularly if an outbreak were to occur during the breeding season. Further, the economic benefit of protective and suppressive vaccination strategies was greater if an outbreak was to occur in the breeding season (August to January), as opposed to outbreaks occurring from February to July. Nevertheless, these strategies could be prioritised based on both the reduced outbreak size, in terms of duration and number of infected properties [8] and the reduced cost to stakeholders. For example, while implementing the protective vaccination strategy was less economically efficient if it were applied in the non-breeding season, it was epidemiologically efficient compared to the baseline strategy as it shortened the overall length of the outbreak, resulting in fewer vaccinated and infected horses. A shorter outbreak will allow the more rapid resumption of normal activity, allowing for more wide-reaching benefits than have been simulated within the current framework. In contrast, the targeted strategy, where only horses involved in the racing industry were vaccinated, would be of greater cost than the baseline and not reduce the duration or size of the outbreak markedly. The targeted strategy could allow the movement of vaccinated horses to race meetings and between stud farms, a possibility that was not considered in the epidemiologic model [8] or within the economic simulation.

Overall, the framework identified here provides benefits for multiple stakeholders within the equine industry. The breeding sector would experience its greatest losses if an EI incursion were to occur during the breeding season, due to a loss of breeding opportunities, live foals born and decreased number of horses sold at sales. Many of these horses are produced for sale to international, rather than domestic markets [28], to buyers that would seek other purchasing opportunities if horses were not available in NZ. Under the framework examined here, the racing sector and trainers would gain the most benefit from any control strategy due to the immediacy of the possible stop and resumption of normal activity. These benefits to this sector are two-fold. Firstly, the vaccination of horses reduces the outbreak duration, whereby normal racing business can resume more quickly. Secondly, regardless of control strategy and if compensation were provided in a similar manner to Australia [32], trainers would be the beneficiaries of this compensation. While compensation is a large cost within the model framework, this model does not account for loss to trainers in stakes earnings, losses to gambling income in the domestic market, or the secondary economy created by racehorses including race-day hospitality, feed merchants, farriers and transportation companies. It is likely that in the event of an outbreak, the focus of domestic gambling will change to other areas, including domestic greyhounds and sports betting or a move to racing from overseas, and will be likely to return to domestic racing, albeit possibly as a lower level, when this resumes. However, the short-term loss of income in stakes money may mean some trainers, jockeys and drivers leave the industry during or shortly after an EI outbreak. Further, it was assumed once EI was eradicated, that the revenue from racing would resume immediately at pre-outbreak levels. This would be unlikely as horses will have lost training opportunities during the outbreak, and as such may not have the fitness required to compete in a race, as many racehorses are transported to training facilities or racecourses for daily training. Consequently, the model provides an underestimate of the costs by not including stakes money or a potential delay in resumption of racing to pre-outbreak levels.

The complete cancellation of Thoroughbred and/or Standardbred sales was not considered here. Instead, it was assumed that sales would be postponed, until either all participating horses were vaccinated or the outbreak was over, rather than cancelled. For yearling sales, which occur at the end of January and beginning of February for Thoroughbreds and Standardbreds, respectively, this assumes that the preparation of horses would still be able to continue during an outbreak for most stud farms, if the outbreak began during the breeding season. Based on the sensitivity analysis conducted in the current study, if sales income decreased by a further 20% from what had previously been modelled, there was little effect on the overall loss caused by the outbreak. However, the B/C ratio of the suppressive vaccination strategy increased by 14%, making this strategy more economically appealing if the impact on sales has been underestimated.

Similar to sales income, the loss of breeding income through the reduction in mares served and foals born would probably be an underestimate of the effect of an EI outbreak in NZ. In both countries, Standardbreds are mated using artificial insemination, while Thoroughbred mares are not [11, 34]. Therefore, during the breeding season the majority of Thoroughbred mares will travel to the stallion [9]. In Australia, Thoroughbred mares outside of the affected states that were destined for service by a stallion in an affected state could have been sent to an alternative local stallion. In contrast, if an outbreak were to occur in NZ, all of NZ would be subject to control strategies and all business activity would halt, leading to a greater potential impact in NZ. The current study modelled the loss of breeding income, based on the decrease in mares served in Australia during and after the EI outbreak in 2007 where the number of Thoroughbred mares served decreased by 8%, whereas the number of Standardbred mares served in affected states decreased by 29%. This finding was unexpected due to the use of assisted technologies in the Standardbred industry, suggesting that the number of Thoroughbred mares served is probably an underestimate of the decrease in the affected states of QLD and NSW and was masked by mares that were retained and therefore mated in unaffected states. Additionally, the larger impact on Standardbred breeding may be due to veterinary resources being redirected to outbreak control rather than to providing their normal services. Sensitivity analysis identified little change in the total cost of an outbreak or the B/C ratio of an outbreak where protective or suppressive vaccination strategies were employed in scenarios where the breeding income decreased by 20%.

One limitation of the current study is the lack of information about the available number of horses in NZ. As the NZ government does not require the registration of horses, the true total number of horses in NZ is unknown. Census data from agricultural businesses keeping horses would not have included all horses in the population, as not all properties where horses were kept would be registered as agricultural businesses. Another study has estimated the horse population to be 120,000 based on information stored in the AgriBase database [33]; however, inaccuracies in this database have previously been reported [20]. Population size and density are important considerations for implementing effective infectious disease control strategies. However, the sensitivity analysis determined that increasing the size of the population did not alter the underlying economic return or effectiveness of the protective or suppressive vaccination strategies, in terms of the total cost to control an outbreak or B/C ratio.

In the CBA reported here, the baseline ‘without’ strategy included a minimum level of outbreak control. This may not be the true ‘without’ scenario considered by decision makers in the event of an EI outbreak. Instead, it is possible that the option to do nothing is considered and therefore no control is undertaken. In this case, the costs of incursion response and compensation would not occur. However, if no control were implemented the outbreak would likely affect more horses for a longer period of time than was currently considered. This would have a greater economic impact on horse welfare and the racing and breeding industries. There would also be long-term implications of allowing EI to become endemic in NZ. Consequently, the ‘without’ scenario explored here would underestimate the cost of a true no control ‘without’ model, whereby underestimating the cost of alternative vaccination strategies. Similarly, actual daily cost of an incursion response (without vaccination) as modelled in the ‘without’ strategies, with a reduction in cost after the first 70 days, may also an underestimate of the true cost of mounting a response to an exotic disease. If the cost of an incursion response were to rise, the benefit of mounting a response that would shorten the outbreak duration would be make these strategies more viable.

While a rare occurrence, infectious disease outbreaks in horses have previously been shown to be costly to the economy of the affected country [4, 7]. For horses involved in the racing industry, and to a lesser extent horses produced for elite sporting competition, commercial value is determined by a subjective measure of performance rather than by a more objective measure, e.g. production (meat, wool, eggs, or milk). Consequently, per head cost assessments are difficult to ascertain for an economic analysis on the scale reported here. While the racing industry contributes 1% of NZ gross domestic product [28], the full value that is added to the economy by the equestrian industry, including non-racing horse owners, would be challenging to quantify as while horses can be a commercial entity, they sit in an unusual niche between companion animals and production livestock. Based on the information available regarding the NZ equine population, most horses would be considered to be kept for recreational or competitive use, animals that would have a value more similar to companion animals [20]. Matheson and Akoorie (33) estimated that sports horses in NZ (those kept for recreational or competitive purposes) contributes $1 billion annually into the economy. The current study has not considered non-racing horses, because in the event of an outbreak most participants in this sector will be more concerned with the social impact of the outbreak, the social impact of illness or the loss of the horse, and the loss of recreation or competitive opportunities, rather than the loss of business activity or income. However, it is acknowledged that the sport horse sector does have commercial breeding and trading activities [35] that would be affected by an EI outbreak.

It was assumed that the economic shock caused by an EI incursion would only occur for the duration of the outbreak or immediately post-eradication, and that this immediate shock under each scenario would be the most important consideration for policy makers. Therefore, multiple year discounting was not applied. However, due to the length of the racehorse production cycle, it would be expected that an outbreak in or around the breeding season would have a longer-term effect on the industry. The production cycle is relative long (three to five years), therefore a decrease in breeding, number of services, foals born, sales and young horses entering training due to an EI outbreak would have an on-going impact. While there may be in an increased value of horses, particularly sales horses, to account for reduced supply, this did not appear to be the case in Australia, where a drop in prices was also seen. As there are already concerns regarding the size and viability of the domestic racing market [36], any reduction in available product may have an impact on the long-term viability of domestic racing. This would require further exploration.

## Conclusions

The outbreak of EI in Australia in 2007 and the closeness and similarity of the two equine industries, forced NZ decision makers to consider the possible implications of EI to the NZ equine industry. The implementation of suppressive or protective vaccination strategies, particularly if an outbreak were to occur during the breeding season, was found to be economically favourable when compared with the baseline strategy. This study has identified that multiple industry stakeholders, particularly those in the racing sector, will benefit from the implementation of vaccination strategies. In some scenarios, these benefits were disproportionate to the cost of an incursion response. The study will support policy development to prioritise strategies and key decision makers—industry, the government and individual horse owners—can now focus the debate regarding user-pays eradication programmes.

## Abbreviations

B: Benefit
B/C ratio: benefit-cost ratio
CBA: Cost-benefit analysis
CV: Vaccination cost
EI: Equine Influenza
NB: net benefit
NSW: New South Wales
NZ: New Zealand
QLD: Queensland
